# Astrocytic IP_3_/Ca^2+^ Signaling Modulates Theta Rhythm and REM Sleep

**DOI:** 10.3389/fncir.2017.00003

**Published:** 2017-01-23

**Authors:** Jeannine Foley, Tamara Blutstein, SoYoung Lee, Christophe Erneux, Michael M. Halassa, Philip Haydon

**Affiliations:** ^1^Department of Neuroscience, Tufts University, BostonMA, USA; ^2^Institut de Recherche Interdisciplinaire en Biologie Humaine et Moléculaire, Université Libre de BruxellesBrussels, Belgium; ^3^Departments of Psychiatry, Neuroscience and Physiology, Neuroscience Institute, New York University, New YorkNY, USA

**Keywords:** EEG, mouse model, REM sleep, NREM sleep, sleep homeostasis, sleep deprivation, astrocyte, Ca^2+^ signaling

## Abstract

Rapid eye movement (REM) sleep onset is triggered by disinhibition of cholinergic neurons in the pons. During REM sleep, the brain exhibits prominent activity in the 5–8 Hz (theta) frequency range. How REM sleep onset and theta waves are regulated is poorly understood. Astrocytes, a non-neuronal cell type in the brain, respond to cholinergic signals by elevating their intracellular Ca^2+^ concentration. The goal of this study was to assess the sleep architecture of mice with attenuated IP_3_ mediated Ca^2+^ signaling in astrocytes. Vigilance states and cortical electroencephalograph power were measured in wild type mice and mice with attenuated IP_3_/Ca^2+^ signaling. Attenuating IP_3_/Ca^2+^ signaling specifically in astrocytes caused mice to spend more time in REM sleep and enter this state more frequently during their inactive phase. These mice also exhibited greater power in the theta frequency range. These data suggest a role for astrocytic IP_3_/Ca^2+^ signaling in modulating REM sleep and the associated physiological state of the cortex.

## Introduction

The major aspects of sleep architecture are conserved from rodent to human. Each vigilance state: rapid eye movement (REM) or paradoxical sleep, non-REM (NREM) sleep, and wake can be distinguished by neuronal synchronization measured by electroencephalography (EEG) and the level of movement detected by electromyography (EMG). During wake, the EEG is characterized by low amplitude, desynchronized activity. The EEG shows increased synchrony during NREM sleep and is dominated by slow wave activity (SWA) in the 0.5–4 Hz range. REM sleep is similar to wake, with a desynchronized EEG but prominent hippocampal theta activity in the 5–8 Hz frequency range (Platt and Riedel, 2011).

Upon natural transition into REM sleep, cholinergic projection neurons in the pons become disinhibited and drive theta oscillations in the hippocampus (McCarley and Massaquoi, 1992; Buzsàki, 2002; McCarley, 2007). Furthermore, experimental activation of pontine neurons using optogenetics (Van Dort et al., 2015) or cholinergic agonists (Bezzi et al., 1998) increases time spent in REM sleep. Interestingly, Hippocampal astrocytes express cholinergic receptors that elicit calcium transients when stimulated (Shen and Yakel, 2012).

Intracellular Ca^2+^ transients in astrocytes mainly occur via release from internal stores upon activation of the inositol triphosphate receptor 2 (IP_3_R2). The extent to which these Ca^2+^ signals provide physiological output is a major focus of current research and is incompletely understood (Khakh and McCarthy, 2015). Genetic deletion of IP_3_R2 does not alter homosynaptic plasticity (Bonder and McCarthy, 2014; Petravicz et al., 2014); however, these Ca^2+^ stores contribute to heterosynaptic, cholinergic-mediated plasticity and performance in the Morris water maze (Takata et al., 2011).

We have previously demonstrated that SNARE-mediated release of gliotransmitters from astrocytes is necessary for sleep homeostasis (Halassa et al., 2009; Schmitt et al., 2012). Considering that Ca^2+^ signaling is required for neurotransmission, and that there is extensive evidence that it is required for gliotransmission (Porter and McCarthy, 1997; Araque et al., 1998; Buzsàki, 2002; Hua et al., 2004; Kreft et al., 2004; Chen et al., 2005; Pryazhnikov and Khiroug, 2008; Paukert et al., 2014), we asked whether we could replicate the dnSNARE phenotype of impaired sleep homeostasis in a mouse model of disrupted astrocytic IP_3_/Ca^2+^ signaling.

We overexpressed a venus tagged IP_3_ 5’phosphatase (VIPP) transgene selectively in astrocytes to enhance the metabolism of IP_3_ to IP_2_ and attenuate IP_3_-mediated Ca^2+^ signaling in astrocytes. We found that VIPP expressing mice had increased REM sleep and theta power, suggesting a previously unrecognized and unique role for astrocytic IP_3_/Ca^2+^ signaling in neuromodulation. Moreover, these results demonstrate the importance of the astrocyte, a glial cell sub-type, in the control of the generation of sleep states and brain rhythms.

## Materials and Methods

### Animals

All procedures were in strict accordance with the National Institutes of Health *Guide for the Care and Use of Laboratory Animals* and were approved by Tufts University and Institutional Animal Care and Use Committees. All animals were housed on a 12 h light/dark cycle and were given standard chow and water *ad libitum*.

The VIPP mouse line was created by fusing the type I Ins(1,4,5)P_3_ 5′-phosphatase (IPP) construct to the Venus construct and co-expressing the Lac-Z reporter (De Smedt et al., 1997). The original IPP construct in pcDNA3 (De Smedt et al., 1997) was released by BamHI/XbaI digestion and cloned into pUC19 cloning vector. The coding region of Venus (Evanko and Haydon, 2005) was PCR amplified, digested with BamHI/NcoI, and ligated in-frame into the corresponding sites of IPP in pUC19. For experiments involving cultured astrocytes, the VIPP fragment was cloned into the expression vector pcDNA3.1. To make the tetO-transgenic vector, the VIPP fragment was excised from pcDNA3.1 using BamHI (blunted)/XbaI, and the ligated into StyI (blunted)/SpeI digested pTg1-tetO vector. The pTg1-tetO-plasmid has been described elsewhere (Pascual et al., 2005). TetO-VIPP and tetO-LacZ were digested to remove bacterial sequences and co-injected into C57BL/6J fertilized zygotes. Founders were identified by PCR, and subsequently crossed with GFAP-tTA animals (Pascual et al., 2005). All breedings were performed with 40 mg/kg doxycycline (Dox, Bioserv, Frenchtown, NJ, USA) in the food, and offspring were maintained on Dox until weaning to suppress transgene expression during embryonic life and post-natal development. VIPP or littermate control C57BL/6J mice were used.

### Slice Preparation

Horizontal hippocampal slices (300–330 μm) from 6 to 10-week-old mice were prepared as described previously (Pascual et al., 2005). Briefly, the brain was rapidly removed and chilled with cold (4°C) artificial cerebrospinal fluid (ACSF) of the following composition (in mM): 124 NaCl, 3.1 KCl, 2 MgCl_2_, 1 CaCl_2_, 26 NaHCO_3_, 1 NaH_2_PO_4_, 10 glucose, 1 Na-pyruvate, and 0.6 ascorbic acid (pH 7.4 adjusted with 95% O_2_, 5% CO_2_) or 85 NaCl, 2.5 KCl, 1.25 NaH_2_PO_4_, 0.5 CaCl_2_, 4 MgCl_2_, 25 NaHCO_3_, 75 sucrose, 25 glucose, and 0.5 ascorbic acid (pH 7.4 with 95% O_2_, 5% CO_2_). The brain was then cut with a Leica VT 1000S vibratome and CA3 to CA1 connections were severed. Slices were incubated at 30–32°C for 1 h recovery and were recorded at 32–34°C.

### Ca^2+^ Imaging in Brain Slices

A multicell bolus loading was performed as previously described (Li et al., 2003). Rhod-2/AM, was dissolved in 20% pluronic acid in DMSO to yield a concentration of 20 mM. For cell loading, this solution was diluted 1/100 with an injection solution containing (in mM) 150 NaCl, 2.5 KCl, 10 HEPES, pH 7.4. An injection pipette (1–2 MΩ) was filled with this solution and inserted into stratum radiatum of CA1 region, and ejected using positive pressure (1 min, 70 kPa; Picospritzer NPI, Germany). One hour later images were acquired using a Q-imaging cooled CCD camera and ImagePro software.

### Slice Stimulation

Wild type and VIPP brain slices were isolated and responses to stimulation were quantified as delta fluorescence, expressed as Δ*F*/*F* = (*F*_1_-*F*_0_)/*F*_0_, where *F*_0_ and *F*_1_ are the value of the fluorescence in the ROI at rest and the given time point, respectively. Slices were stimulated with either 100 μM ATP application or electrical stimulation of the Schaffer collateral fibers. Stimulation pulses were elicited using a 125 μm concentric Pt-Ir electrode. Either 1 s of 100 Hz or five theta burst trains were used to stimulate slices. Theta burst stimulation was 100 Hz for 40 ms with 200 ms intervals. Recordings of astrocyte responses to stimulation were made in area CA1.

### Astrocyte Culture, Transfection, and Ca^2+^ Imaging

Cortical astrocyte cultures were prepared from 1 to 2-day-old mice. Cells were grown in modified Eagle’s medium containing 10% fetal bovine serum, 2 mM L-glutamine, 40 mM D-glucose, 14 mM sodium bicarbonate, 1% sodium pyruvate, and 1% penicillin/streptomycin in 95% air/5% humidified CO_2_. Confluent cultures were shaken overnight followed by a media change and a subsequent round of shaking. After the final shaking, adherent cells were dissociated using 0.25% trypsin and 1 mM EDTA, and replated on glass coverslips. Cells were used after 2–4 days in culture.

Astrocytes were transfected using Fugene (Roche, Indianapolis, IN, USA) and 24–72 h post-transfection, astrocytes cotransfected with IPP and DsRed were loaded with Fluo-4 by incubation in Fluo-4/AM, and Ca^2+^ imaging was performed on the stage of an inverted microscope. Astrocytes transfected with VIPP were loaded with Rhod-2 by incubation in Rhod-2/AM and Ca^2+^ imaging was performed in a similar fashion. Ins(1,4,5)P_3_ 5′-phosphatase activity at 1 μM substrate level was performed on VIPP transfected COS-7 cells as described previously (Terunuma et al., 2008).

### Surgery

Mice aged 8–12 weeks were anesthetized with isoflurane and placed into a stereotaxic frame. Electroencephalogram (EEG) head implants (Pinnacle Technology, Inc.) were placed as previously described (Clasadonte et al., 2014). After surgery, mice were subcutaneously injected with buprenorphine (0.08 mg/kg) and lactated Ringer’s solution, and fed moistened rodent food. After 5 days of postoperative recovery, lightweight recording cables were connected to the head implants and mice were placed in Circular Plexiglas cages (Pinnacle Technology, Inc.) and acclimated for a week.

### Data Acquisition

Electroencephalography/electromyography activity was continuously monitored for 48 h. EEG/EMG activity starting at Zeitgeber time (ZT) 0 was measured for 24 h (baseline), followed by 6 h of sleep deprivation enforced by gentle handling. EEG/EMG activity was monitored during the 6 h of sleep deprivation and 18 h of recovery sleep. During data acquisition, EEG signals were high pass filtered at 0.5 Hz and low pass filtered at 40 Hz. EMG signals were high pass filtered at 0.5 Hz and low pass filtered at 100 Hz. The amplifier system (Pinnacle Technology, Inc.), sampled at 250 Hz with a PAL 8400 data acquisition system (Pinnacle Technology, Inc.).

### Vigilance State Scoring and Analysis

Sleep stages were scored visually based on 4 s epochs by a trained experimenter using SleepSign for Animal software (Kissei Comtec). Wakefulness consisted of low-amplitude, high-frequency EEG, and high EMG activity; REM sleep consisted of low-amplitude EEG activity with prominent 5–8 Hz frequency and low EMG activity; NREM sleep consisted of high-amplitude, low-frequency EEG with little EMG modulation. Time spent in each state was expressed as a percentage of the total recording time in 1 h time bins. EEG power spectra of consecutive 4 s epochs [fast Fourier transform (FFT) routine; Hanning window] were calculated. The EEG power during NREM sleep from 0.5 to 1.5 Hz was defined as low frequency slow wave activity (lf-SWA) and was used as a quantitative measure of sleep pressure and homeostatic sleep drive (Franken et al., 2001; Cirelli et al., 2005). The EEG power of lf-SWA during NREM sleep was used to assess hour-by-hour sleep power. Hour by hour lf-SWA was normalized to the last 4 h of the baseline day. The EEG power from 5.0 to 8.0 Hz was defined as theta activity and was used as a measure of theta power. To compare theta power across genotypes, raw data were normalized to 0.5 to 1.5 Hz frequency range. Normalization in this range was chosen because it best represented the raw data and there were no differences in power between wild type and VIPP mice in this frequency range.

### Tissue Processing and Immunohistochemistry

Eight to ten week old animals were cardiac perfused with phosphate buffered saline (PBS) and paraformaldehyde (PFA). The brain was extracted, post-fixed in PFA for 1 h at 4°C, placed overnight in 30% sucrose and frozen at -80°C. Coronal sections (40 μm) were cut, washed with PBS, and permeabilized with 0.1% Triton X-100, 5% horse or goat serum in PBS. Unless otherwise indicated the antibodies used were from Millipore, Temecula, CA, USA. We used mouse NeuN antibody, 1:1000, rabbit NG2 antibody, 1:1000, rabbit GFAP antibody (Sigma, St. Louis, MO, USA), 1:1000, and rabbit Iba1 antibody (Wako Chemicals, Richmond, VA, USA), 1:500. Secondary antibodies conjugated to either Alexa 633 or 568 were used. Sections were mounted and visualized on either Fluoview 1000 confocal microscope (Olympus, Center Valley, PA, USA) or A1 confocal microscope (Nikon, Tokyo, Japan). Venus fluorescence was detected at 515 nm.

### Statistics

For all experiments except sleep studies, comparisons between two groups were conducted with Student’s *t*-test. For sleep studies, comparisons between two groups were made using a Mann–Whitney *U* test. Groups with multiple data points were compared using two-way repeated measures ANOVA, followed by a Tukey’s *post hoc* multiple-comparisons test. Statistical significance was defined as *p* < 0.05. Data are presented as mean ± SEM unless otherwise stated.

## Results

### Astrocyte-Selective Expression of VIPP Attenuates Astrocytic Ca^2+^ Signals

To determine the importance of astrocytic IP_3_/Ca^2+^ signals, we used molecular genetics to selectively impair IP_3_/Ca^2+^ signaling in astrocytes. IP_3_ is metabolized through type I Ins(1,4,5)P_3_ 5′-phosphatase (IPP) activity (De Smedt et al., 1997). We transiently transfected cultured astrocytes to express IPP and demonstrate that it attenuates agonist-induced Ca^2+^ signaling in these cells (**Supplementary Figure S1A**; 83 ± 2% reduction of the Ca^2+^ signal, *n* = 10 coverslips). To generate a fluorescent variant of IPP for use in transgenic animals, we tagged the N-terminal of IPP with the YFP variant Venus. Transient transfection and over expression of Venus-IPP fusion protein (VIPP) attenuated Ca^2+^ signaling in cultured astrocytes (**Supplementary Figure S1B**; 67 ± 3% reduction of the Ca^2+^ signal, *n* = 6 coverslips). When the transgenic vector was transfected into COS-7 cells, anti-GFP blotting of protein lysates revealed a band corresponding to the predicted size of the Venus-IPP fusion protein (**Supplementary Figure S1C**), and biochemical analysis showed a 40-fold increase in IPP activity (**Supplementary Figure S1D**).

**FIGURE 1:**
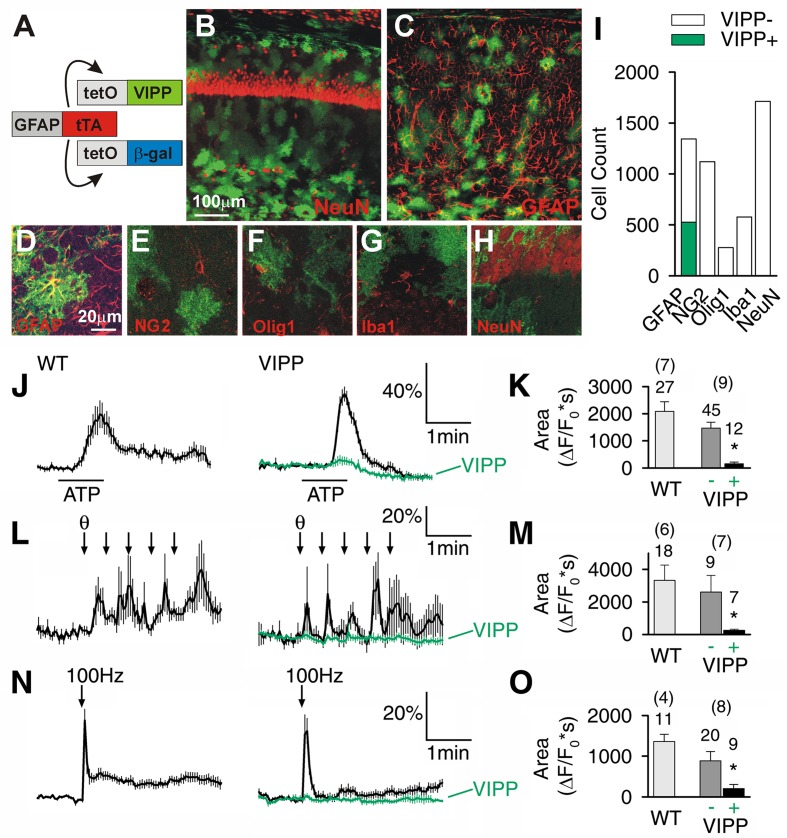
**Astrocyte-specific expression of venus tagged IP_3_ 5′phosphatase (VIPP) attenuates Ca^2+^ signaling in hippocampal CA1 astrocytes.** **(A)** Cartoon depicting the GFAP promoter driving the expression of VIPP and β-gal. **(B,C)** Top view confocal images (20× 0.7NA objective) of hippocampal CA1-region showing VIPP expression and CA1 neurons (NeuN) and astrocytes (GFAP). **(D–H)** High magnification single confocal planes (60× 1.4NA objective) showing VIPP colocalizes with GFAP **(D)**, but not with NG2 **(E)**, Olig1 **(F)** Iba1 **(G)** or NeuN **(H)**. **(I)** Quantification of **D–H**. *n* = 6 animals for each staining. **(J,K)** Astrocytic Ca^2+^ responses in response to 100 μM ATP **(J,K)**, five theta trains **(L,M)** or 100 Hz, 1 s tetanic stimulation **(N,O)** are significantly attenuated by expression of VIPP (green). Quantitation **(K,M,O)** is shown as the integral of Δ*F*/*F*_0_ signal for 2 min after ATP application (*t*-test, ^∗^*p* < 0.0016 in **K**; *p* < 0.025 in **M**; *p* < 0.006 in **O**), for 3 min after theta burst stimulation and 3 min following tetanic stimulation of the Schaffer collaterals. Number of tested cells and tested slices (in parenthesis) are indicated.

We generated astrocyte-specific transgenic animals using the Tet-Off system (**Figure 1A**) (Pascual et al., 2005; Halassa et al., 2009) to allow conditional expression of VIPP in astrocytes (VIPP mice). TetO.VIPP and tetO.β-gal were co-injected into C57BL/6J fertilized zygotes to generate tetO.VIPP founder lines, which were screened for transgene expression by being crossed with GFAP.tTA mice (**Figure 1A**) (Pascual et al., 2005; Halassa et al., 2009). Bigenic offspring of one line showed abundant Venus fluorescence when examined by confocal microscopy (**Figures 1B–H**). Double-labeling experiments demonstrated that VIPP was selectively expressed in astrocytes. Venus fluorescence was observed in 39% of hippocampal area CA1 GFAP positive astrocytes (*n* = 527 out of 1343 from six animals; **Figures 1C,D,I**), but was never detected in neurons (NeuN; **Figures 1B,H**), NG2-glia (NG2; **Figure 1E**), oligodendrocytes (olig1; **Figure 1F**), or microglia (Iba1; **Figure 1G**). In addition to hippocampal VIPP expression, we also observed transgene expression in the brainstem (**Supplementary Figure S3A**), and minimal expression in the cortex. Double labeling with anti-NeuN never showed transgene expression in neurons in any brain region.

We studied stimulus-induced astrocytic Ca^2+^ signaling in the hippocampus, a brain region with prominent theta activity. We found astrocytic Ca^2+^ was attenuated in slices derived from VIPP mice. The time-integral of the Ca^2+^ response was attenuated from a wild-type value of 2083 ± 360 Δ*F*/*F*_0_^∗^s to 156 ± 61 Δ*F*/*F*_0_^∗^s in astrocytes visually confirmed to express VIPP (*p* < 0.0016, *t*-test) in response to exogenous application of ATP (100 μM). Astrocytes with undetectable VIPP exhibited Ca^2+^ signals that were not significantly different from wild-type values (**Figures 1J,K**). Similarly, synaptic activity-dependent recruitment of astrocytic Ca^2+^ signals was attenuated in VIPP expressing astrocytes (**Figures 1L–O**). Ca^2+^ signals in VIPP expressing astrocytes were attenuated from 3327 ± 940 Δ*F*/*F*_0_^∗^s to 253 ± 70 Δ*F*/*F*_0_^∗^s (*p* < 0.025, *t*-test; **Figures 1L,M**) following five trains of theta burst stimulation. The ability of VIPP to attenuate astrocytic Ca^2+^ signals was not stimulus-specific since the response to 100 Hz tetanic trains was also significantly reduced in VIPP expressing astrocytes (*p* < 0.006, *t*-test; **Figures 1N,O**).

### VIPP Mice Exhibit Increased REM Sleep

**FIGURE 2:**
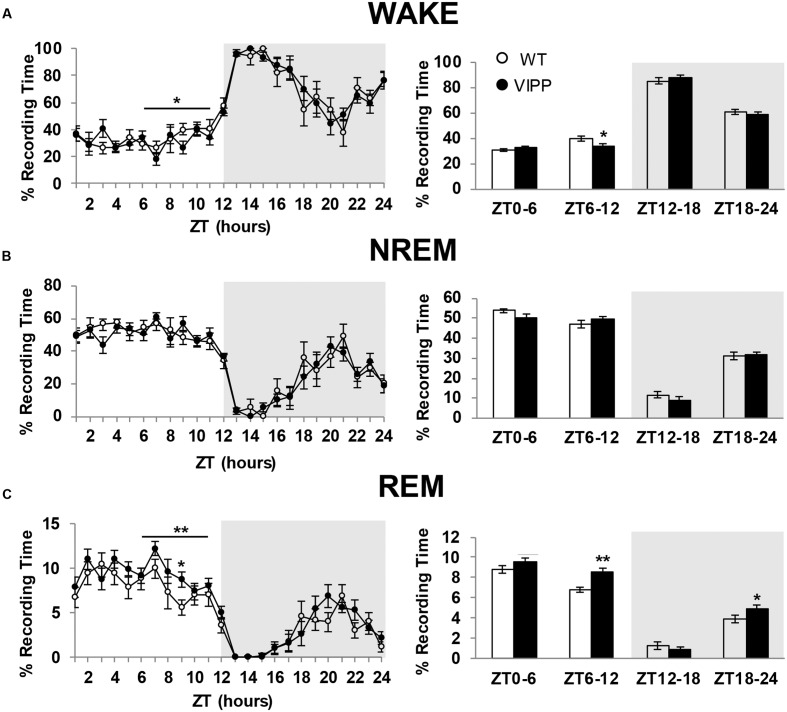
**Astrocytic VIPP expression increases time spent in rapid eye movement (REM) sleep and decreases time spent in wake.** **(A)** Percent recording time spent in wakefulness. VIPP mice spent significantly less time awake during ZT 6-12 (left: ^∗^*p* < 0.05, two-way RM ANOVA; right: ^∗^*p* < 0.05, Mann–Whitney *U* test). **(B)** Percent recording time spent in NREM sleep. Astrocytic VIPP and wild type mice spent the same amount of time in NREM sleep (two-way RM ANOVA: n.s.). **(C)** Percent recording time spent in REM sleep. VIPP mice spent significantly more time in REM sleep during the second half of the light cycle and the second half of the dark cycle (left: ^∗∗^*p* < 0.01, two-way RM ANOVA, ZT9: Tukey’s *post hoc* test: *p* < 0.05; right: ^∗^*p* < 0.05, ^∗∗^*p* < 0.01, Mann–Whitney *U* test). Data are represented as mean ± SEM.

**FIGURE 3:**
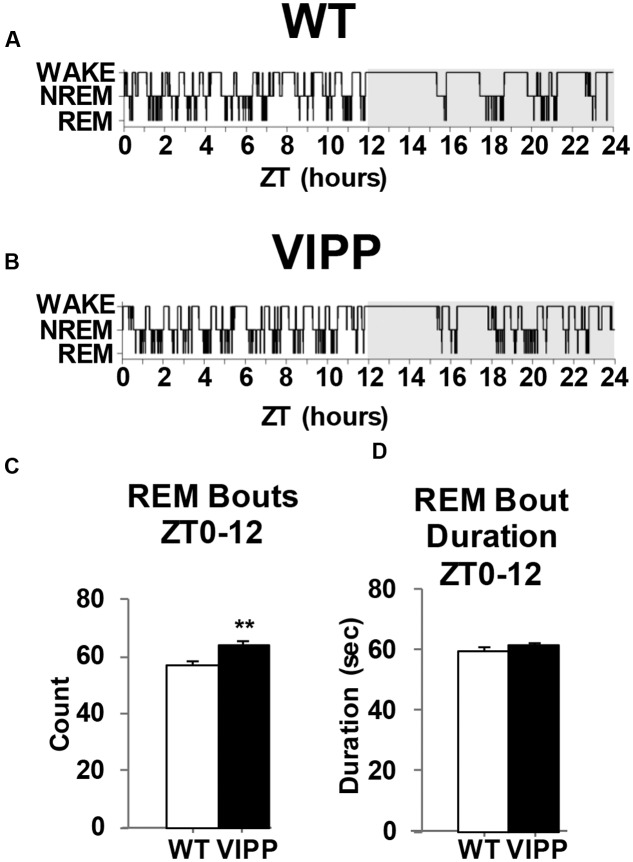
**Mice that overexpress IP_3_ 5′-phosphatase in astrocytes have increased REM sleep bouts.** **(A)** Representative example of a hypnogram from a wild type and **(B)** a VIPP mouse. **(C)** VIPP mice entered REM sleep more frequently than wild type mice during the light phase. **(D)** Wild type and VIPP mice had similar REM bout durations during the light phase [values represent mean and standard error (SEM) for each group, ^∗∗^*p* < 0.01, Mann–Whitney *U* test].

To examine the importance of astrocytic IP_3_/Ca^2+^ signaling in sleep regulation, we asked whether VIPP expression altered the duration of each vigilance state. The percent time spent in REM sleep was increased in VIPP expressing mice, compared to wild type mice (**Figure 2**; VIPP, *n* = 12; WT, *n* = 8). This was most apparent during the light phase (subjective nighttime), and corresponded to an increase in number of REM bouts (represented in **Figures 3A,B** and quantified in **Figure 3C**, VIPP = 63.75 ± 1.6, wild type = 56.5 ± 2.0; *p* < 0.01). However, REM bout duration was unchanged in VIPP mice (**Figure 3D**). Mice spent significantly more time in REM sleep during the second half of the light phase and the second half of the dark phase (**Figure 2C**, ZT6-12; VIPP = 8.6 ± 0.4%, wild type = 6.8 ± 0.3%; *p* < 0.01; ZT18-24; VIPP = 4.9 ± 0.4%, wild type = 3.9 ± 0.4%; *p* < 0.05). During the second half of the light phase, when VIPP mice spent a far greater percentage of time in REM sleep, compared to wild type mice, they spent significantly less time awake (**Figure 2A**, ZT6-12; VIPP = 34.5 ± 1.3%, wild type = 39.8 ± 2.0%; *p* < 0.05). Throughout the light/dark cycle, VIPP and wild type mice spent similar time in NREM (**Figure 2B**). These data suggest that astrocyte IP_3_/Ca^2+^ signaling specifically modulates the REM phase of sleep, particularly in the later portion of the inactive period.

### Astrocytic IP_3_/Ca^2+^ Signaling Modulates REM But Not NREM Sleep Homeostasis

Astrocytes modulate plasticity and cortical oscillations as well as sleep homeostasis (Fellin et al., 2009; Halassa et al., 2009; Lee et al., 2014). Using genetically modified mice that have attenuated gliotransmission, the dnSNARE mouse, it was shown that the gliotransmitter adenosine (derived from ATP) is necessary for the accumulation of sleep pressure (Halassa et al., 2009). Given that Ca^2+^ signaling can contribute to gliotransmission (Porter and McCarthy, 1997; Araque et al., 1998; Buzsàki, 2002; Hua et al., 2004; Kreft et al., 2004; Chen et al., 2005; Pryazhnikov and Khiroug, 2008; Paukert et al., 2014), we asked whether we could reproduce the dnSNARE phenotype of impaired sleep homeostasis by disrupting astrocytic IP_3_/Ca^2+^ signaling. We compared baseline SWA and SWA following sleep deprivation in VIPP and wild type littermate mice. VIPP mice neither exhibited alterations in SWA (0.5–4 Hz) nor low frequency-SWA (lf-SWA, 0.5–1.5 Hz) either in baseline conditions or following sleep deprivation compared to wild type littermates (**Supplementary Figure S2**) suggesting that IP_3_/Ca^2+^ signaling does not modulate NREM sleep homeostasis.

**FIGURE 4:**
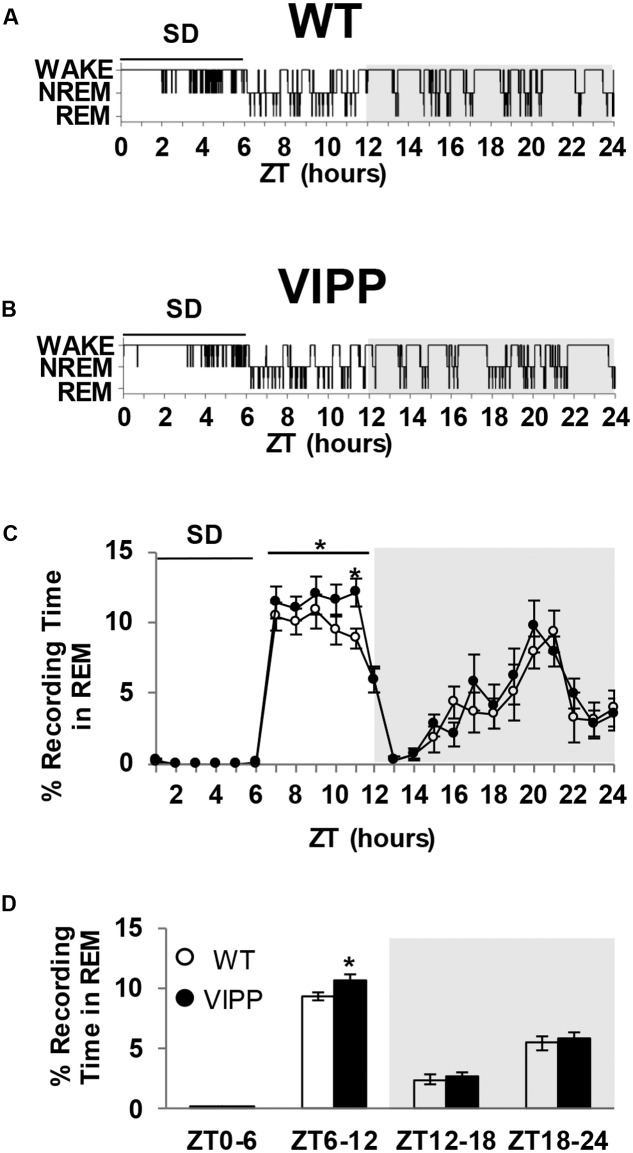
**Enforced wake enhances REM rebound in VIPP mice to a greater degree than wild type mice.** **(A)** Representative example of a wild type mouse hypnogram during (ZT0-6) and after SD (ZT6-24). **(B)** Representative example of a VIPP mouse hypnogram during (ZT0-6) and after SD (ZT6-24). **(C)** Percent time spent in REM sleep during the 6 h of sleep deprivation and subsequent recovery period. VIPP mice spent significantly more time in REM sleep during the recovery period compared to wild type mice (ZT6-12: two-way RM ANOVA: ^∗^*p* < 0.05; ZT11: Tukey’s *post hoc* test: ^∗^*p* < 0.05). **(D)** Average percent recording time spent in REM sleep. VIPP mice spend significantly more time in REM sleep after SD, compared to wild type mice (ZT6-12: ^∗^*p* < 0.05, Mann–Whitney *U* test).

The magnitude of the homeostatic increase in SWA delta power that occurs with prolonged wake is positively correlated with the duration of subsequent NREM sleep (McCarley, 2007). REM sleep is also homeostatically regulated; REM-specific sleep deprivation results in REM specific rebound sleep (Kitka et al., 2009). However, the regulation of REM sleep homeostasis is poorly understood. VIPP mice show enhanced REM rebound sleep after 6 h of sleep deprivation, compared to wild type mice (**Figure 4**). The greatest difference is seen in the later phase of the light cycle, during ZT10-11 (**Figure 4C**; *p* < 0.05, Tukey’s *post hoc* test). These data indicate that astrocyte IP_3_/Ca^2+^ signaling modulates the homeostatic increase in REM sleep after SD.

### VIPP Mice Exhibit Increased Theta Power

**FIGURE 5:**
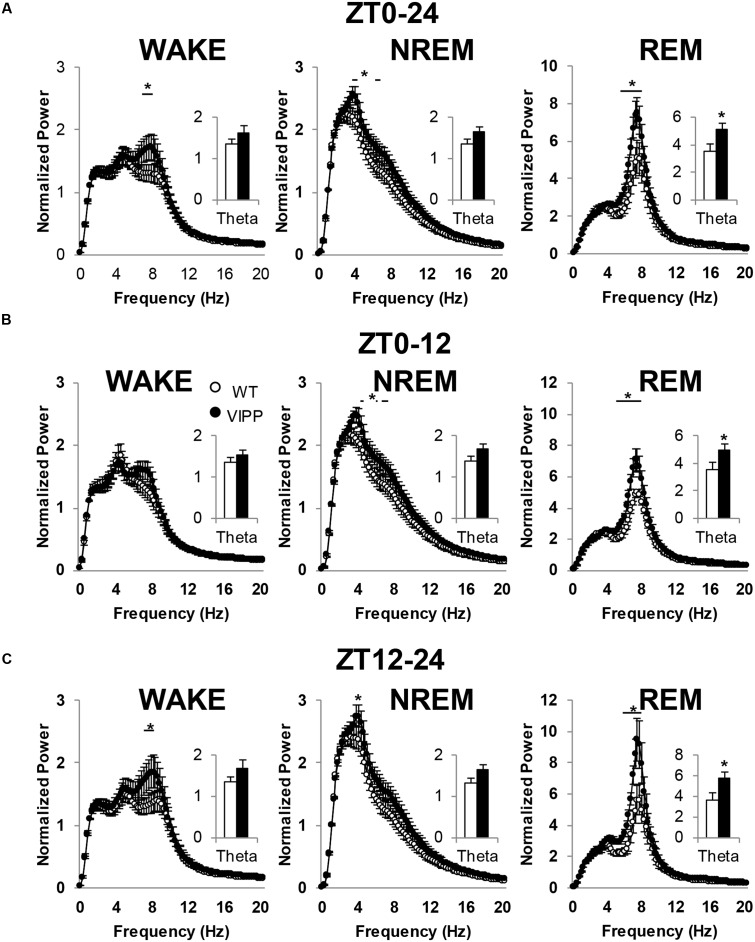
**Astrocytic VIPP mice exhibit increased theta power compared to wild type mice.** **(A)** EEG power over the entire 24-h recording period; inset shows average theta (5–8 Hz) power. VIPP mice had increased theta power across all vigilance states (WAKE: two-way RM ANOVA: ^∗^*p* < 0.05; inset: n.s.; NREM: two-way RM ANOVA: ^∗^*p* < 0.05; inset: n.s.; REM: two-way RM ANOVA: ^∗^*p* < 0.05; inset: Mann–Whitney *U* test, ^∗^*p* < 0.05). **(B)** Light cycle FFT data representation of EEG power. Theta power is enhanced in VIPP mice during NREM and REM sleep (WAKE: two-way RM ANOVA: n.s.; inset: n.s.; NREM: two-way RM ANOVA: ^∗^*p* < 0.05; inset: n.s.; REM: two-way RM ANOVA: ^∗^*p* < 0.05; inset, Mann–Whitney *U* test: ^∗^*p* < 0.05). **(C)** Dark cycle FFT data representation of EEG power. Theta power was enhanced in VIPP mice during WAKE, NREM, and REM. (WAKE: two-way RM ANOVA: ^∗^*p* < 0.05; inset: n.s.; NREM: two-way RM ANOVA: ^∗^*p* < 0.05; inset: n.s.; REM: two-way RM ANOVA: ^∗^*p* < 0.05; inset, Mann–Whitney *U* test: ^∗^*p* < 0.05). Data are represented as mean ± SEM.

We next examined power spectra associated with each vigilance state. We found enhanced EEG theta power in VIPP mice, compared to wild type mice (**Figure 5**). This effect was present during wakefulness, NREM and REM sleep. However, the most robust effect occurred during REM sleep, where theta is the dominant frequency in the power spectrum; although, theta is also a prominent frequency in wakefulness during active stimulus processing (Colgin, 2013). When a power analysis was performed on the entire 24 h baseline day, VIPP mice exhibited enhanced theta power (**Figure 5A**; WAKE: *p* < 0.05 at 7.1–8.1 Hz; NREM: *p* < 0.05 at 3.6–4.1, 5.8–6.3 Hz; REM: *p* < 0.05 at 5.9–8.1 Hz). When overall theta power (5–8 Hz) was averaged and compared across genotypes, VIPP mice expressed significantly increased power during REM sleep (*p* < 0.05; wild type: 3.6 ± 0.5; VIPP: 5.1 ± 0.4) but not during other vigilance states.

To assess a time-of-day effect on theta power, the baseline day was broken down into the light and dark phase and power spectra were analyzed for each vigilance state within these time bins. In both the light and dark phase, average theta power was significantly increased during REM sleep (**Figures 5B,C**). During the light phase, VIPP mice exhibited enhanced power within the theta frequency range across both NREM and REM sleep (**Figure 5B**; WAKE: NS; NREM: *p* < 0.05 at 3.9–4.1, 5.3, 5.8–6.3 Hz; REM: *p* < 0.05 at 5.4–8.1 Hz). When theta power was averaged across the entire 5–8 Hz frequency range, an increase in average theta power was observed in VIPP mice during REM sleep only (inset: wild type: 3.5 ± 0.5; VIPP: 5.0 ± 0.4; *p* < 0.05). During the dark phase, VIPP mice exhibited enhanced theta power across all vigilance states (**Figure 5C**; WAKE: *p* < 0.05 at 7.1–8.1 Hz; NREM: *p* < 0.05 at 3.9 Hz; REM: *p* < 0.05 at 6.1–8.1 Hz), but when averaged across frequencies, VIPP mice had significantly greater theta power during REM sleep only (wild type: 3.7 ± 0.6; VIPP: 5.8 ± 0.6; *p* < 0.05). These data suggest that astrocyte IP_3_/Ca^2+^ signaling modulates theta power, especially during REM sleep.

## Discussion

Gliotransmission and astrocytic Ca^2+^ signaling affect neuronal activity in many ways (Araque et al., 2014). The present study examined the behavioral effect of attenuated astrocytic IP_3_ dependent Ca^2+^ signaling throughout the brain. The major conclusions from this study are that attenuated astrocytic IP_3_/Ca^2+^ signaling leads to a modulation of REM sleep and theta rhythm power. When this astrocytic signaling pathway is impaired, mice spend more time in REM sleep due to an increased frequency of REM bouts with no alteration in the duration of individual events. Unique from the role of astrocyte gliotransmission in NREM sleep homeostasis, impairment of IP_3_/Ca^2+^ signaling in astrocytes does not attenuate the homeostatic increase in lf-SWA after sleep deprivation. However, since minimal transgene expression was found in the basal forebrain and cortex in VIPP mice, these different observations may result from a lack of VIPP expression in these regions that are important in sleep homeostasis. Additionally, these observations do not rule out potential contributions of other Ca^2+^ sources including Ca^2+^ influx pathways in contributing to gliotransmission in sleep homeostasis.

Mice with attenuated IP_3_/Ca^2+^ signaling specifically in astrocytes spent more time in REM sleep than wild type mice (**Figure 2C**). This was likely due to the fact that these VIPP expressing mice entered REM sleep more frequently (**Figure 3C**). VIPP mice had enhanced theta power, particularly during REM sleep (**Figure 5**). These data suggest a role for astrocyte IP_3_/Ca^2+^ signaling in dampening theta power, perhaps via cholinergic signaling in the hippocampus.

Our understanding of how cholinergic input modulates neuronal and astrocytic function is incomplete (McQuiston, 2014). However, given that astrocytes respond to cholinergic input in the hippocampus (Costa e Silva, 2006; Pabst et al., 2016), future studies will determine whether the enhancement of REM sleep and theta power in VIPP mice occurs through cholinergic signaling in the hippocampus. The pons is of particular importance in the generation of REM sleep and is also a prominent source of cholinergic signaling. Future experiments to assess brain region specific effects of the VIPP transgene in response to cholinergic agonists will provide evidence of where VIPP expression is required for a REM phenotype. Furthermore, considering that theta power is most prominent during REM sleep, testing a temporal effect of astrocyte Ca^2+^ perturbation using targeted optogenetics may lead to a more mechanistic understanding of astrocytic Ca^2+^ signaling in theta rhythm and REM sleep regulation. Assuming the mechanism involves Ca^2+^ dependent exocytosis of gliotransmitter, the next goal will be to determine which gliotransmitters regulate this pathway.

Our findings that attenuating IP_3_-dependent Ca^2+^ signaling in astrocytes increases REM sleep and theta power conflict with a previous report that knockout of the IP_3_R2 does not alter sleep architecture in mice (Cao et al., 2013). The difference in results may be attributed to the inducible nature of our genetic manipulation. In the Itpr2^-/-^ mice used by Cao et al. (2013), the IP_3_R2 is deleted throughout life, which may allow for developmental adaptations to the absence of this receptor. However, our method of inducible overexpression of IPP allows for acute manipulation and analysis of astrocyte mediated IP_3_Ca^2+^ signaling. Additionally, because we targeted the metabolism of IP_3_ in our study it is possible that some of the different results arise from differences in IP_3_ metabolism between Itpr2^-/-^ and VIPP mice. Although reasons for the discrepancy in results using different mice remain uncertain, because we found that several different parameters of REM sleep were perturbed following VIPP expression we are confident that astrocytic VIPP expression leads to REM phenotypes. For example, we observed increased theta power during REM sleep, an increase in time spent in REM sleep that results from an increase in the number of bouts of REM sleep and finally we observed that rebound REM sleep was enhanced following sleep deprivation. Taken together, this is the first study to show that *in vivo* manipulation of astrocyte IP_3_/Ca^2+^ signaling directly affects sleep behavior.

We used the TetO system to induce VIPP expression in astrocytes upon weaning. In this system, TetO is activated by tTA (**Figure 1A**). Doxycycline (included in the diet) inhibits this interaction and prevents transgene expression until doxycycline is withdrawn from the diet. Doxycycline is known to exert off-target effects (Ahler et al., 2013) in addition to regulating transgene expression. Thus, all of our control comparisons were made between wild type and transgenic animals in the same doxycycline condition.

Recent publications challenge the validity of the TetO system as a tool for astrocyte-specific expression (Fujita et al., 2014). Fujita et al. (2014) report findings of neuronal expression in dnSNARE mice, also driven by the TetO system, although numerous other laboratories have been unable to reproduce this anomaly. The reasons for this discrepancy are unclear but may result from genetic drift of mice maintained in different facilities and underscore the importance of repeated characterization of lines of mice before making conclusions about cell specificity. In our studies using the tetO.VIPP animals, we have never observed neuronal expression. We see extremely rare VIPP expression in the cortex and only in astrocytes, never in neurons. We report abundant VIPP expression in the brainstem (**Supplementary Figure S3A**). This expression does not co-localize with the neuronal marker, NeuN (**Supplementary Figure S3B**).

The homeostatic drive to sleep is characterized by increased SWA power, or sleep pressure (McCarley, 2007). We have previously shown that astrocyte gliotransmission contributes to the accumulation of sleep pressure (Halassa et al., 2009). Here, in contrast, we show that SWA sleep pressure is unaltered in mice with attenuated astrocyte IP_3_/Ca^2+^ signaling (**Supplementary Figure S2**). NREM sleep and delta power are attenuated in dnSNARE mice, but remain intact in VIPP mice, suggesting astrocyte SNARE-mediated gliotransmission modulates sleep through a mechanism that is independent of IP_3_/Ca^2+^ signaling. Furthermore, VIPP mice spend more time in REM sleep and dnSNARE mice do not exhibit a REM sleep phenotype (Halassa et al., 2009), suggesting IP_3_/Ca^2+^ signaling regulates REM sleep independently of SNARE-mediated gliotransmission. These data suggest that there are distinct astrocytic processes that regulate different aspects of sleep and cortical function in general. Indeed, in a recent study tetanus toxin disruption of vesicular mediated release from astrocytes decreased gamma (25–80 Hz) frequency activity (Lee et al., 2014). Taken together, these studies demonstrate a previously unrecognized complexity to the signals that astrocytes can utilize to modulate neuronal networks and rhythm generation.

Mood disorders are associated with increased cholinergic to aminergic neurotransmitter levels (Costa e Silva, 2006). Accordingly, depressed individuals spend more time in REM sleep (Steiger and Kimura, 2010). Treatment with antidepressants decreases time spent in REM and discontinuation of medication causes rebound REM sleep. Given that astrocytes respond to cholinergic and aminergic stimulation by elevating intracellular Ca^2+^ (Costa e Silva, 2006; Ding et al., 2013; Paukert et al., 2014), and we found that mice with attenuated astrocytic IP_3_/Ca^2+^ signaling show increased REM sleep, further investigation of this pathway may provide insight into the link between depression and REM sleep.

## Author Contributions

JF and PH: wrote manuscript. JF: EEG data analysis. TB: EEG data collection and analysis. SL: Ca^2+^ imaging data collection and analysis. CE: provided IPP construct and performed biochemistry. MH: conceived the use of IPP to suppress Ca^2+^ in astrocytes, designed and generated the VIPP fusion, performed functional characterization with Ca^2+^ imaging in culture, generated transgenic vectors and performed initial characterization of founder mice.

## Conflict of Interest Statement

PH is the president and founder of GliaCure, Inc. All the other authors declare that the research was conducted in the absence of any commercial or financial relationships that could be construed as a potential conflict of interest.
