# Supplementary material for: Astrocytic IP3/Ca2+ Signaling Modulates Theta Rhythm and REM Sleep
Source: Front Neural Circuits. 2017 Jan 23;11:3. doi: 10.3389/fncir.2017.00003 (PMC5253379; doi:10.3389/fncir.2017.00003)
Supplement: Supplementary file 1 [file Data_Sheet_1.DOCX]

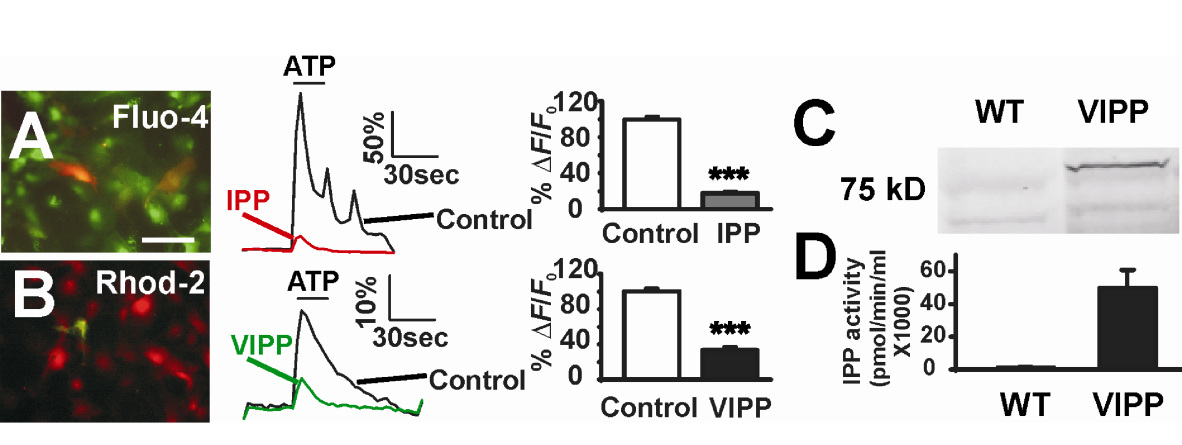


**Supplementary Figure S1. Transfection of VIPP reduces the amplitude of ATP-induced astrocytic Ca^2+^ signals. (A)** Left, cultured astrocytes co-transfected with IPP and DsRed (scale bar = 100 μm) exhibit attenuated Fluo-4 response following 100 μM ATP application (middle). Right, average Ca^2+^ response (n = 10 cultures, ***, P < 0.0001, t-test). **(B)** Following VIPP transfection cultured astrocytes experiments similar to **(A)** were performed using Rhod-2 (n = 6 cultures, ***, P < 0.0001, t-test). **(C)** anti-GFP blotting reveals a band at 75 kD with VIPP transfected COS-7 cell lysates (D). These lysates show a 40-fold increase of IPP biochemical activity.


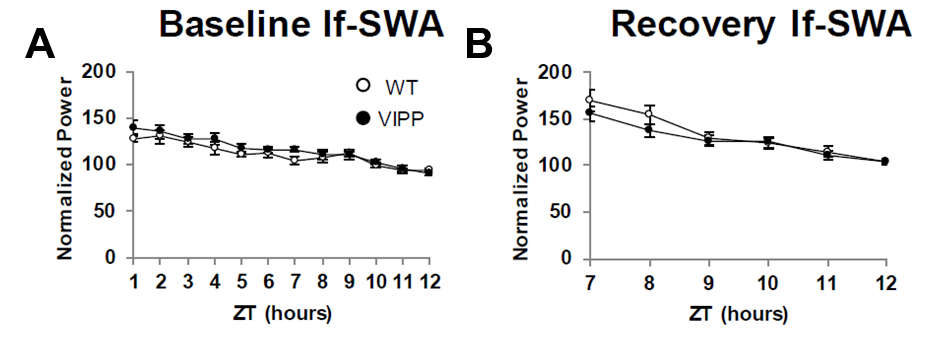


**Supplemental Figure S2. Astrocytic VIPP mice exhibit normal SWA at baseline and after SD.** **(A)** Normalized lf-SWA in wild type and VIPP mice on the baseline day. VIPP and wild type mice had similar levels of lf-SWA on the baseline day from ZT0-12 (Two-way RM ANOVA; p=0.28). **(B)** Normalized lf-SWA in wild type and VIPP mice after SD. VIPP and wild type mice had similar levels of lf-SWA after recovery from 6hr SD (Two-way RM ANOVA; p=0.48).

**
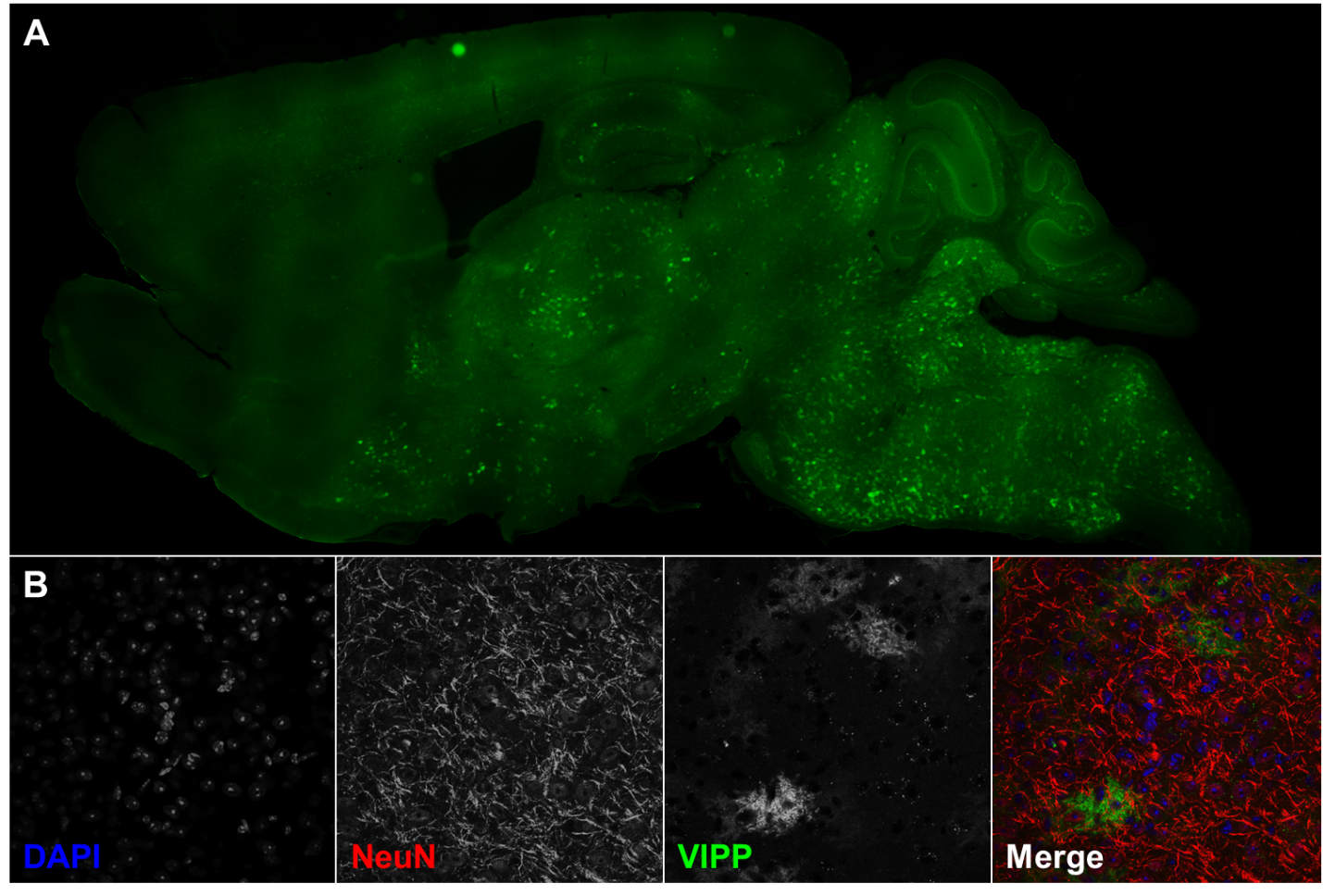
**

**Supplemental Figure S3. VIPP expression is widespread and does not co-express with NeuN in the brainstem. (A)** Brain wide VIPP expression. (B) Greyscale images of DAPI labeled nuclei, NeuN fluorescently labeled cells, VIPP fluorescence, and a merge of the three channels on the far right. Images were taken from the brainstem at 60x.
